# Mitotic Spindle Attachment to the Holocentric Chromosomes of *Cuscuta europaea* Does Not Correlate With the Distribution of CENH3 Chromatin

**DOI:** 10.3389/fpls.2019.01799

**Published:** 2020-01-24

**Authors:** Ludmila Oliveira, Pavel Neumann, Tae-Soo Jang, Sonja Klemme, Veit Schubert, Andrea Koblížková, Andreas Houben, Jiří Macas

**Affiliations:** ^1^ Biology Centre, Czech Academy of Sciences, Institute of Plant Molecular Biology, České Budějovice, Czechia; ^2^ Department of Breeding Research, Leibniz Institute of Plant Genetics and Crop Plant Research, Gatersleben, Germany

**Keywords:** centromere, kinetochore, CENH3, holocentric chromosomes, repetitive DNA analysis, satellite DNA, *Cuscuta*

## Abstract

The centromere is the region on a chromosome where the kinetochore assembles and spindle microtubules attach during mitosis and meiosis. In the vast majority of eukaryotes, the centromere position is determined epigenetically by the presence of the centromere-specific histone H3 variant CENH3. In species with monocentric chromosomes, CENH3 is confined to a single chromosomal region corresponding to the primary constriction on metaphase chromosomes. By contrast, in holocentrics, CENH3 (and thus centromere activity) is distributed along the entire chromosome length. Here, we report a unique pattern of CENH3 distribution in the holocentric plant *Cuscuta europaea*. This species expressed two major variants of CENH3, both of which were deposited into one to three discrete regions per chromosome, whereas the rest of the chromatin appeared to be devoid of CENH3. The two CENH3 variants fully co-localized, and their immunodetection signals overlapped with the positions of DAPI-positive heterochromatic bands containing the highly amplified satellite repeat CUS-TR24. This CENH3 distribution pattern contrasted with the distribution of the mitotic spindle microtubules, which attached at uniform density along the entire chromosome length. This distribution of spindle attachment sites proves the holocentric nature of *C. europaea* chromosomes and also suggests that, in this species, CENH3 either lost its function or acts in parallel to an additional CENH3-free mechanism of kinetochore positioning.

## Introduction

Centromeres are chromosomal regions that facilitate faithful chromosome segregation during cell division. This is achieved by providing an anchor point for assembly of a kinetochore, a protein complex that connects centromeric chromatin to the spindle microtubules (Cheeseman, 2014). Most higher plant species possess monocentric chromosomes, in which the mitotic spindle binds to a single region on each chromosome that is discernible as the primary constriction on condensed metaphase chromosomes. On the other hand, holocentric chromosomes lack primary constrictions and have spindle binding sites distributed along almost the entire chromosome length. Holocentric taxa have a broad phylogenetic distribution, including various groups of nematodes, arthropods, and plants (Melters et al., 2012). In flowering plants, they represent a minor fraction, including, for example, families Juncaceae (Bozek et al., 2012), Cyperaceae (Luceño et al., 1998; Roalson et al., 2007; Håkansson, 2010), Droseraceae (Kolodin et al., 2018); genus *Chionographis* [Liliaceae; (Tanaka and Tanaka, 1979)]; and some species from the genus *Cuscuta* (Pazy and Plitmann, 1994; Pazy and Plitmann, 1995; Pazy and Plitmann, 2002). Because holocentric taxa are often embedded within broader phylogenetic lineages possessing monocentric chromosomes, it is thought that holocentric chromosome organization originated from the monocentric format and that this transition occurred independently in multiple phylogenetic lineages (Melters et al., 2012). However, the factors that induced this transition and its mechanisms are currently unknown.

In most organisms, centromeres are determined epigenetically by the presence of the centromere-specific histone variant CENH3 (Allshire and Karpen, 2008). In monocentric chromosomes, CENH3 is confined to the primary constrictions, whereas in holocentrics it is distributed along the chromosomes concurrently with spindle attachment sites. In exceptional cases, CENH3 genes have been lost altogether in some holocentric organisms (Drinnenberg and Akiyoshi, 2017). These include four lineages of insects in which the loss of CENH3 correlated with the transition to holocentricity, suggesting a causal relationship between the two events (Drinnenberg et al., 2014). In plants, only a few holocentric species have been studied in detail, including representatives of families Juncaceae (*Luzula*) and Cyperaceae (*Rhynchospora*). Chromosomes in these species are characterized by a longitudinal centromere groove that correlates with the presence of CENH3 and attachment of spindle microtubules (Nagaki et al., 2005; Heckmann et al., 2011; Marques et al., 2015; Wanner et al., 2015). In *Rhynchospora pubera*, specific repeats are associated with CENH3-containing chromatin, containing the satellite DNA family TYBA; two different TYBA-containing repeats, TCR1 and TCR2; and the LTR-retrotransposon CRRh (Marques et al., 2015; Ribeiro et al., 2017). By contrast, no centromere-specific repeats have been identified in *Luzula elegans* (Heckmann et al., 2013).

Investigation of the changes associated with the transition from monocentric to holocentric chromosome organization is, in theory, most informative when comparing phylogenetically closely related species that differ in centromere type. The best-documented such case is genus *Cuscuta*, in which holocentric chromosomes have been identified in particular species using classical cytogenetics techniques (Pazy and Plitmann, 1991; Pazy and Plitmann, 1994; Pazy and Plitmann, 1995; Pazy and Plitmann, 2002). Evidence for the existence of holocentric chromosomes includes 1) absence of primary constrictions, 2) orientation of chromosomes parallel to the equatorial plane during mitotic metaphase and anaphase, 3) tolerance of chromosomes to fragmentation, and 4) inverted meiosis. The genus includes about 200 species of obligatory parasitic plants that depend on their host plants for water and nutrients (García et al., 2014). Three distinct phylogenetic lineages exist within the genus, corresponding to subgenera *Monogynella*, *Grammica*, and *Cuscuta*. The first two subgenera include monocentric species, whereas subgenus *Cuscuta* presumably consists entirely of holocentrics (Pazy and Plitmann, 1994; Pazy and Plitmann, 1995; Pazy and Plitmann, 2002; McNeal et al., 2007; García et al., 2014). However, these species have never been studied in detail using molecular techniques.

In this work, we performed a detailed molecular-cytogenetic characterization of *Cuscuta europaea* as a representative holocentric *Cuscuta* species. To investigate centromeric chromatin, we identified *CENH3* genes in *C. europaea*, as well as in two representatives of monocentric species, and investigated the distribution of CENH3 chromatin and microtubule attachment sites along mitotic chromosomes. In addition, we characterized repetitive DNA sequences and mapped their distribution along chromosomes with regard to the patterns of CENH3 distribution.

## Materials and Methods

### Plant Materials

Seeds of *C. europaea* (serial number: 0101147) were obtained from the Royal Botanic Garden (Ardingly, UK). Seeds of *C. campestris* and *C. japonica* were provided by Dr. Chnar Fathoulla (University of Salahaddin, Kurdistan Region, Iraq) and Dr. Takeshi Furuhashi (RIKEN Center for Sustainable Resource Science, Yokohama, Japan), respectively. To ensure rapid germination, seeds of *C. europaea* were treated in sulfuric acid for 1 h at room temperature, washed three times in distilled water, sterilized in 0.1× SAVO (UNILEVER, Prague, Czech Republic; 1× SAVO contains 4.7% NaOCl) for 20 min, and washed three times in sterile distilled water. Finally, the sterile seeds were germinated on a solid 0.5× Murashige and Skoog medium (Duchefa, Haarlem, the Netherlands) containing 0.8% agar supplemented with 3% sucrose. Seeds of the other two species were abraded with a sandpaper or scalpel and germinated on a damp paper towel in a Petri dish. *Cuscuta* seedlings that were 1.5–2 cm long were transferred onto their host plants: *Urtica dioica* (*C. europaea*), *Ocimum basilicum* (*C. campestris*), and *Pelargonium zonale* (*C. japonica*). All *Cuscuta* species were grown in isolation to prevent their accidental spread.

### RNA Isolation, Sequencing, and Identification of CENH3-Coding Sequences

Total RNA was isolated from shoots and inflorescences of *C. europaea* and *C. campestris* using Trizol reagent (Invitrogen, Carlsbad, CA, USA) and treated with DNase I (Ambion, Austin, TX, USA). Before sequencing, RNA from the two tissues was mixed in a 1:1 ratio and subjected to two rounds of poly-A mRNA subtraction using the Dynabeads mRNA purification kit (Thermo Fisher Scientific, Waltham, MA, USA). RNA sequencing was performed at GATC Biotech AG (Konstanz, Germany) using Illumina technology to produce 50 nt paired-end reads. The data were deposited in the Short Read Archive (SRA; https://www.ncbi.nlm.nih.gov/sra) under accessions ERR3651372 and ERR3651373. Illumina RNA-seq data from *C. japonica* were downloaded from SRA (run accessions DRR021689 and DRR021687). *De novo* transcriptome assemblies for all three *Cuscuta* species were built using Trinity (Grabherr et al., 2011). Contig sequences with similarity to CENH3 were identified using tBLASTn (Altschul, 1997), with query containing a set of CENH3 sequences published previously or downloaded from GenBank. Because the histone fold domain (HFD) at the C-terminus of CENH3 shares relatively high similarity with canonical H3 histones, many of the tBLASTn hits belonged to the latter histone variant. However, CENH3 and H3 could be clearly distinguished because the entire H3 histone sequence is nearly invariant across all eukaryotes, whereas CENH3 histones vary among species and differ from classical H3 histones at the N-terminus. The existence of multiple CENH3 variants in *C. europaea* (CENH3^CEURO-1a^, CENH3^CEURO-1b^, and CENH3^CEURO-2^) and *C. campestris* (CENH3^CCAMP-a^ and CENH3^CCAMP-b^) was confirmed experimentally by RT-PCR and 3'RACE amplification, followed by sequencing of the cloned products. RT-PCR and 3'RACE were performed as described previously (Neumann et al., 2015). First-strand synthesis was performed using the SuperScript III First-Strand Synthesis System for the RT-PCR kit (Thermo Fisher Scientific). Sequences of primers used for reverse transcription and PCR, along with details of the amplification conditions, are provided in **Supplementary Table 1**. Sequences of selected clones representing all CENH3 variants in the two *Cuscuta* species were deposited into GenBank under accession numbers MN625517-MN625524. The CENH3 sequence from *C. japonica* was not verified experimentally because the same sequence was assembled using two different RNA-seq datasets, and the RNA-seq reads did not indicate the presence of multiple variants.

### Analysis of Repetitive DNA Sequences

Genomic DNA used for sequencing was extracted from young shoots of *C. europaea* as described previously (Dellaporta et al., 1983). Shotgun sequencing of the DNA was performed by University of Rochester Genomics Research Center (New York, NY, USA), employing an Illumina platform to generate 100 nt paired-end reads from ~300–500 bp fragment libraries. The sequence data was deposited in SRA under run accession ERR3528104. Repetitive sequences were identified by similarity-based clustering of Illumina paired-end reads using the RepeatExplorer (Novák et al., 2013) and TAREAN (Novák et al., 2017) pipelines. The numbers of analyzed reads were 2,565,388 and 254,708, corresponding to 0.22× and 0.02× genome coverage, respectively. All clusters representing at least 0.01% of the genome were manually checked, and their automated annotation was corrected if needed; finally, the clusters were used to characterize and quantify the most abundant repeats. Genome proportions of the major repeat types (**Supplementary Table 2**) were calculated based on the proportion of reads in individual annotated clusters. Abundances of simple sequence repeats, such as (TAA)n, were calculated using Tandem Repeats Finder (TRF) (Benson, 1999) and TRAP (Sobreira et al., 2006). The input for TRF was prepared by concatenating one million randomly selected reads, each of which was separated by a stretch of 50 Ns.

### *In Situ* Immunodetection and FISH

**Figure 1:**
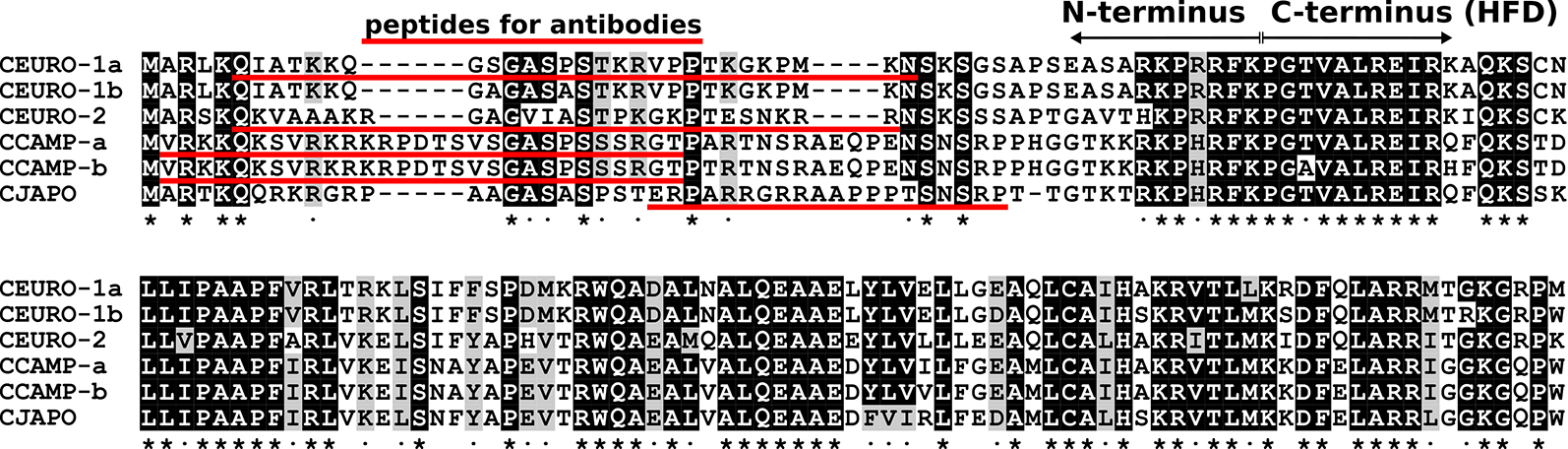
Comparison of CENH3 protein sequences identified in *C. europaea* (CEURO), *C. campestris* (CCAMP), and *C. japonica* (CJAPO). The alignment of CENH3 protein sequences is shaded to highlight invariable sites (black) and sites containing similar amino acid residues (gray). These sites are also marked below the alignment by asterisks and dots, respectively. Note that the sequences are relatively conserved at their C-termini, which contain the histone fold domain (HFD), but are highly divergent at the N-terminus. Sequences of peptides used to produce CENH3-specific antibodies are underlined.

Affinity-purified rabbit polyclonal antibodies against peptides corresponding to N-terminal sequences of individual CENH3 variants were custom-produced by GenScript (Piscataway, NJ, USA; antibodies against CENH3^CEURO-1a^, CENH3^CEURO-2^, and CENH3^CCAMP-a/b^) or Biomatik (Cambridge, Canada; antibody against CENH3^CJAPO^). Peptide sequences are highlighted in **Figure 1**. Because the CENH3^CEURO-1a^ and CENH3^CEURO-1b^ variants differ at only two of 28 amino acid residues of the peptide sequence, the antibody raised primarily to CENH3^CEURO-1a^ is likely to have recognized both CENH3^CEURO-1^ variants. Mouse monoclonal antibody to α-tubulin was purchased from Sigma Aldrich (St. Louis, MO, USA; catalog number: T6199).

*In situ* immunodetection of CENH3 and α-tubulin was done as described by (Neumann et al., 2012; Neumann et al., 2015) with the following modifications. For detection of CENH3, chromosome preparations were made in LB01 lysis buffer (15 mM Tris, 2 mM Na_2_EDTA, 80 mM KCl, 20 mM NaCl, 0.5 mM spermine, 15 mM mercaptoethanol, 0.1% Triton X-100, pH 7.5) by squashing shoot apical meristems fixed in TRIS-fix buffer (4% formaldehyde, 10 mM Tris, 10 mM Na_2_EDTA, 100 mM NaCl, pH 7.5) for 30 min at 10°C (infiltration of the fixative was improved by application of vacuum for the first 5 min), followed by digestion with 2% cellulase ONOZUKA R10 (SERVA Electrophoresis, Heidelberg, Germany) and 2% pectinase (MP Biomedicals, Santa Ana, CA, USA) in PBS buffer for 110 min at 27.4°C. For simultaneous detection of CENH3 and α-tubulin, suspension of chromosomes and nuclei isolated from fixed shoot apical meristems were prepared as described (Neumann et al., 2002), and then were spun on slides using a Hettich centrifuge equipped with cytospin chambers. Rabbit and mouse primary antibodies were detected using goat anti-rabbit Rhodamine Red-X (1:500 dilution; Jackson ImmunoResearch, Suffolk, UK; catalog number: 111-295-144) and goat anti-mouse Alexa Fluor 488 (1:500 dilution; Jackson ImmunoResearch; catalog number: 115-545-166), respectively. For combined detection of CENH3 and CUS-TR24, shoot apical meristems were fixed in 3:1 ethanol:glacial acetic acid for 30 min at 10°C, digested with 2% cellulase and 2% pectinase in PBS for 110 min at 27.4°C, and finally squashed in 45% acetic acid. Immunodetection and FISH were performed in consecutive steps as described (Neumann et al., 2012).

Mitotic chromosomes for FISH experiments were prepared from shoot apical meristems synchronized using ice-cold water for 17 h and fixed in a 3:1 solution of methanol:glacial acetic acid for at least 1 day. The fixed meristems were washed three times for 5 min in distilled water (5 min each), and then incubated in a solution of 2% cellulase and 2% pectinase in PBS for 70 min at 37°C. The samples were then washed carefully with cold distilled water, transferred to a glass slide, and macerated in a drop of cold 3:1 fixative solution (ethanol:glacial acetic acid) using fine-pointed forceps. Finally, the slides were warmed over an alcohol flame, air-dried, and stored at 4°C for up to 3 months. The oligonucleotide probes labeled at the 5' end with fluorescein isothiocyanate (FITC; CUS-TR24) or biotin [CUS-TR2, CUS-TR25, and (TAA)_n_] were purchased from Integrated DNA Technologies (Leuven, Belgium). Fragments of the other repetitive sequences were amplified from genomic DNA of *C. europaea* (CUS-TR65, CUS-TR66, and CUS-TR67) or *Pisum sativum* (5S and 45S rDNA), and were cloned into pCR4-TOPO vector (Thermo Fisher Scientific). Sequences of the clones are provided in **Supplementary Data S1**. These probes were labeled with biotin-16-dUTP (Roche, Mannheim, Germany) using nick translation as described (Karafiátová et al., 2016). FISH was performed according to Macas et al. (2007), with hybridization and washing temperatures adjusted to account for AT/GC content and hybridization stringency allowing for 10%–20% mismatches. Biotin-labeled probes were detected using Streptavidin-Alexa Fluor 488 (Jackson ImmunoResearch) or Streptavidin-Alexa Fluor 568 (Thermo Fisher Scientific). The slides were counterstained with 4′,6-diamidino-2-phenylindole (DAPI) and mounted in Vectashield mounting medium (Vector Laboratories, Burlingame, CA, USA).

### Microscopy

Conventional wide-field fluorescence microscopy was performed using a Zeiss AxioImager.Z2 microscope equipped with an AxioCam 506 mono-color camera. The microscope was also equipped with an Apotome2.0 device for better resolution in the z-axis, which was needed when the images were composed of multiple optical sections. Images were generated using the ZEN 2 blue software (Carl Zeiss GmbH). To analyze the distribution of microtubules along the chromosomes at the super-resolution level (~120 nm using a 488-nm laser), spatial structured illumination microscopy (3D-SIM) was performed using a 63×/1.4 Oil Plan-Apochromat objective on an Elyra PS.1 microscope system, controlled by the ZENblack software (Carl Zeiss GmbH). Images were captured using the 405-, 488-, and 561-nm laser lines for excitation and the appropriate emission filters. Three-dimensional movies were produced from SIM image stacks using the Imaris 8.0 (Bitplane) and ZENblack software.

## Results

Because our strategy for investigating holocentric chromosomes of *C. europaea* required the visualization of CENH3 protein, which is a universal marker of the centromeric chromatin in plants, we first identified CENH3-coding genes in this species. In parallel, we also investigated representatives of two phylogenetic lineages of monocentric *Cuscuta* species, *C. japonica* (subgenus *Monogynella*) and *C. campestris* (subgenus *Grammica*). Putative CENH3 sequences were identified in short-read transcriptomic data and then verified by cloning complete transcripts obtained by RT-PCR and 3'RACE. In monocentric species, we found a single CENH3 sequence in *C. japonica* (CENH3^CJAPO^) and two sequence variants in *C. campestris* (CENH3^CCAMP-a^ and CENH3^CCAMP-b^), which corresponded to their diploid and tetraploid chromosome numbers, respectively. By contrast, the diploid holocentric species *C. europaea* yielded three variants of CENH3 (CENH3^CEURO-1a^, CENH3^CEURO-1b^, and CENH3^CEURO-2^). The CENH3 sequences (**Figure 1**) differed considerably between species, as they did between the CENH3^CEURO-1a/b^ and CENH3^CEURO-2^ variants in *C. europaea* (**Table 1**). We then designed polyclonal antibodies for immunodetection of CENH3 on chromosomes, targeting the N-terminal regions, which were the most variable parts of these proteins (**Figure 1**).

**Table 1:** Pairwise similarities between CENH3 sequences.

	CEURO-1a	CEURO-1b	CEURO-2	CCAMP-a	CCAMP-b	CJAPO
CEURO-1a	–	94.6	65.5	60.1	60.1	54.4
CEURO-1b	96.2/93.8	–	65.5	58.1	58.1	57.8
CEURO-2	48.1/75.0	51.9/72.9	–	61.7	61.7	62.2
CCAMP-a	42.3/69.8	38.5/68.8	37.7/75.0	–	97.5	75.7
CCAMP-b	44.2/68.8	40.4/67.7	39.6/74.0	98.4/96.9	–	73.7
CJAPO	35.3/64.6	39.2/67.7	40.4/74.0	57.1/86.5	55.4/84.4	–

All values are percentages. The percent similarities between entire CENH3 protein sequences are shown above the diagonal, whereas percent similarities between the N- and C-terminal parts are shown below the diagonal (N/C).

**Figure 2:**
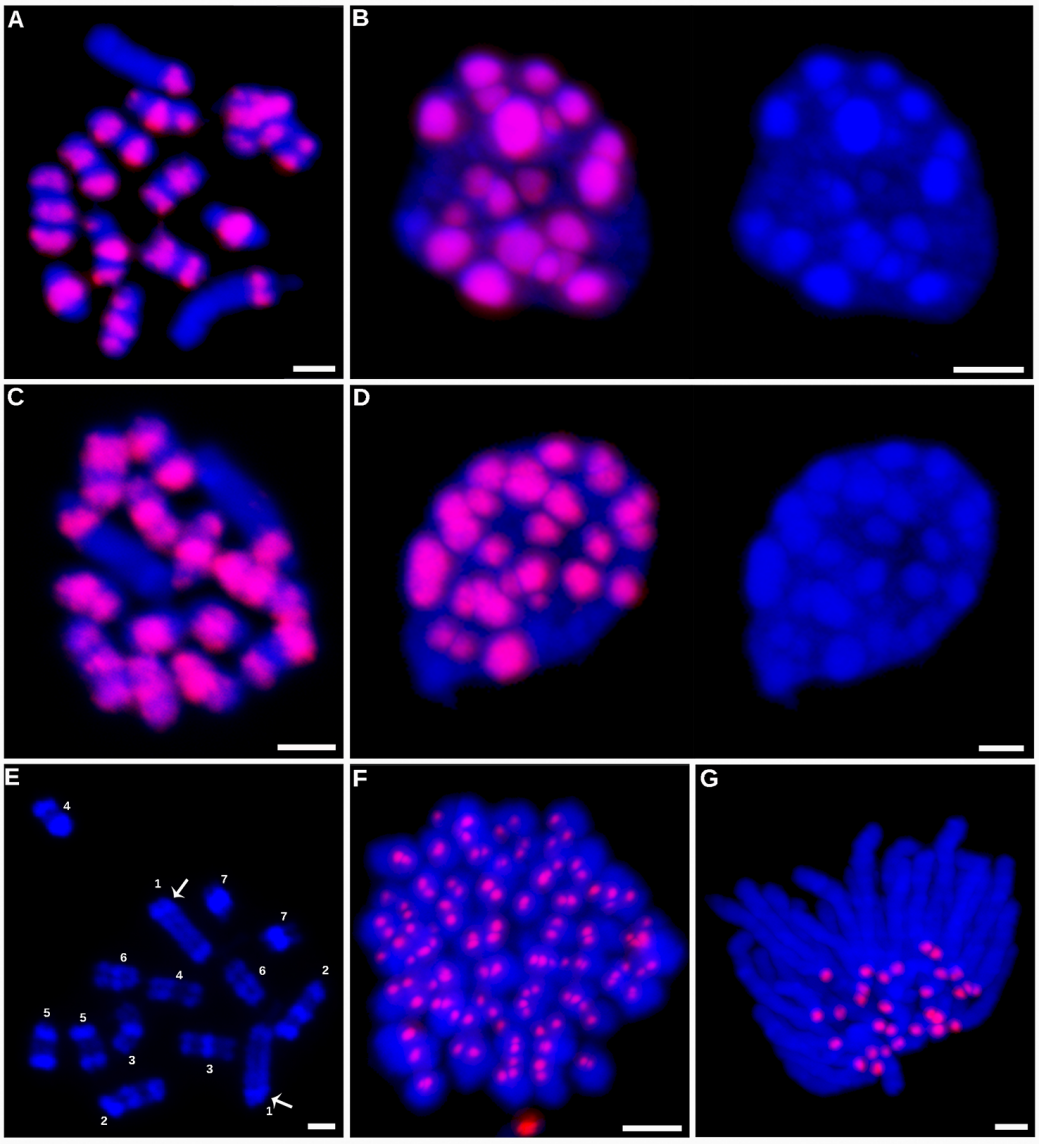
Immunodetection of CENH3 proteins. Immunodetection of CENH3 proteins in *C. europaea* **(A–D).** Detection of the CENH3^CEURO-1a/b^ variant on metaphase chromosomes **(A)** and interphase nucleus **(B)**. Detection of the CENH3^CEURO-2^ variant on metaphase chromosomes **(C)** and interphase nucleus **(D)**. Nuclei in the panels **(B** and **D)** are shown with and without the CENH3 signals to demonstrate correlation between the distribution of CENH3 and DAPI-positive heterochromatin domains. **(E)** Distribution of DAPI-positive heterochromatin domains on *C. europaea* metaphase chromosomes, prepared from 3:1 fixed meristems to achieve better contrast between heterochromatic and euchromatic regions. Distribution of the heterochromatin domains allowed to distinguish all seven chromosome pairs in this species. Arrows mark the heterochromatin band on chromosome 1 that lacked CENH3 signal. **(F)** Detection of CENH3^CCAMP-a/b^ on metaphase chromosomes of *C. campestris*. **(G)** Detection of CENH3^CJAPO^ on anaphase chromosomes of *C. japonica.* CENH3 signals and DAPI-stained chromosomes are shown in red and blue, respectively. Scale bars = 2 µm.

In *in situ* immunodetection, these antibodies revealed unexpected patterns of CENH3 distribution on mitotic metaphase chromosomes of holocentric *C. europaea* (**Figure 2**). Contrary to previously characterized holocentric plant species, in which CENH3 is always distributed in narrow stripes positioned on the lateral sides of the sister chromatids and extending along almost the entire chromosome length, *C. europaea* chromosomes exhibited an uneven labeling, with CENH3 signals concentrated in one to three bands arranged across the chromosome width (**Figures 2A, C**). The number of CENH3 bands did not correlate with chromosome size: the largest chromosome (chromosome 1) of the *C. europaea* karyotype displayed a single subterminal CENH3 band, whereas the smaller chromosomes each had one to three CENH3 bands. The remaining parts of the chromosomes were devoid of detectable CENH3 signal. The immunodetection signals overlapped with DAPI-positive heterochromatic bands, with the exception of one of the two DAPI bands on chromosome 1, which was free of CENH3 signal (**Figures 2A, C, E**). A clear association between CENH3 proteins and heterochromatin was also observed in interphase nuclei (**Figures 2B, D**). The same labeling patterns were obtained using antibodies for both major variants, CENH3^CEURO-1a/b^ and CENH3^CEURO-2^. This atypical distribution of CENH3 raised doubts about the protein's role in kinetochore establishment in holocentric *Cuscuta* species. On the other hand, CENH3 function was maintained in the two monocentric *Cuscuta* species, which both displayed the expected patterns of CENH3 localization in the primary constrictions of mitotic chromosomes (**Figures 2F–G**).

**Figure 3:**
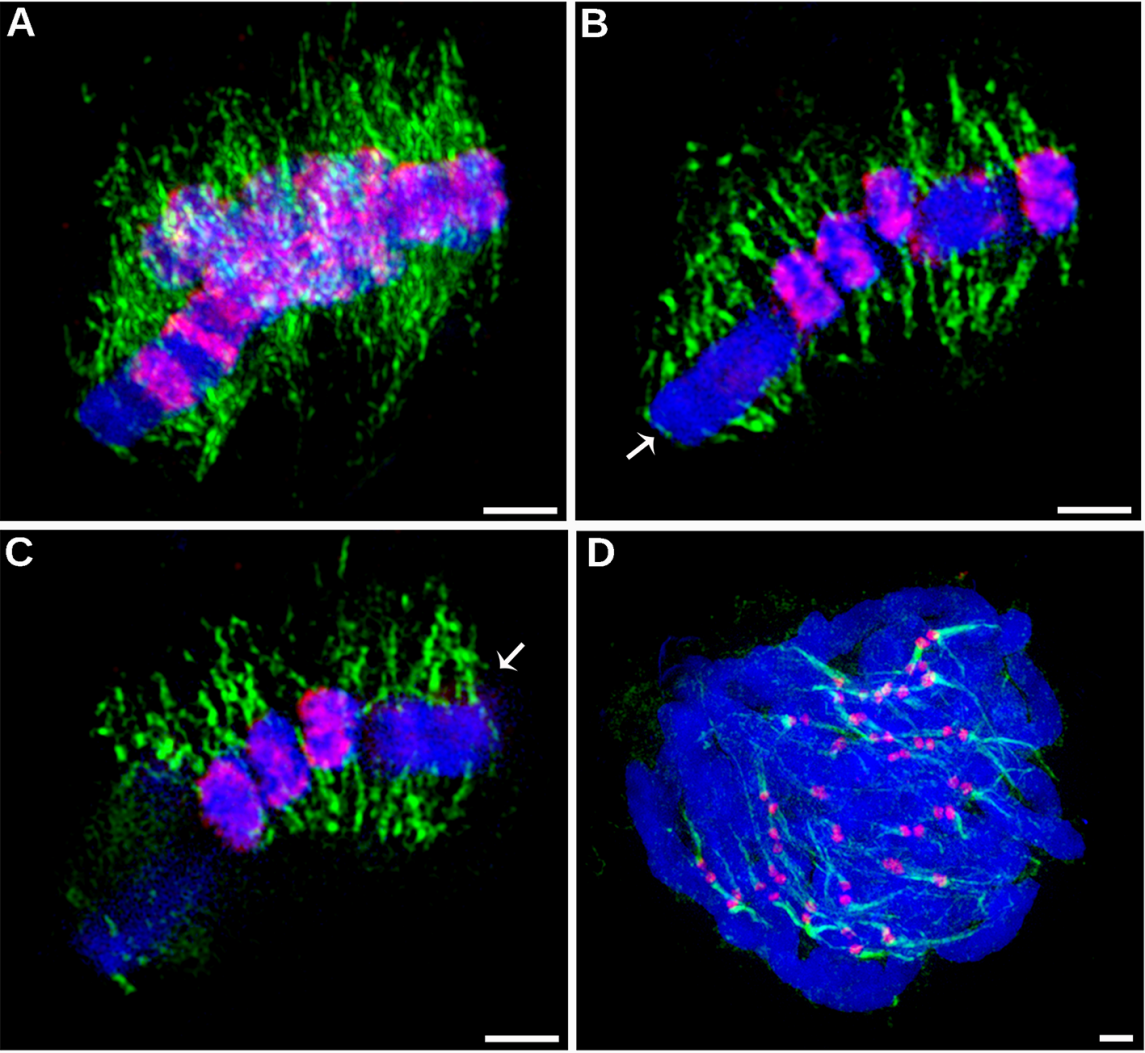
Distribution of CENH3 and spindle attachment sites on chromosomes, investigated by super-resolution structured illumination microscopy (3D-SIM). **(A–C)** Detection of CENH3 (red) and tubulin (green) on metaphase chromosomes of *C. europaea*. The CENH3 was detected using mixed antibodies to CENH3^CEURO-1a/b^ and CENH3^CEURO-2^ (red). Microtubules were detected using α-tubulin antibody (green). **(A)** Maximum-intensity projection image reconstructed from 3D SIM image stack. **(B–C)** Two optical sections selected from the same 3D SIM image stack. They show that microtubules of the mitotic spindle are evenly attached to chromosomes at their poleward sides and along their entire lengths, regardless of the occurrence of CENH3 signals. Arrows mark chromosome 1 which exhibits large CENH3-free region. The full set of optical sections and corresponding projections are also available as **Supplementary Movies 1** and **2**. **(D)** Detection of CENH3 (red) and α-tubulin (green) on metaphase chromosomes of *C. japonica*. Note that microtubules attach to the chromosomes exclusively at CENH3-containing domains. Chromosomes were stained with DAPI (blue). Scale bars = 2 µm.

Prompted by the atypical distribution of CENH3 on *C. europaea* chromosomes, we next investigated the distribution of mitotic spindle attachment sites along the chromosomes. Should CENH3 act as an epigenetic mark of centromeric chromatin, as it does in other plant species, kinetochore formation (and thus the spindle attachment sites) would be expected to co-localize with the CENH3 bands. However, spatial structured illumination microscopy (3D-SIM, super-resolution) revealed that the spindle microtubules visualized by α-tubulin antibody were evenly attached along the entire chromosome length, regardless of the distribution of the CENH3 signals (**Figure 3** and **Supplementary Movies 1** and **2**). This was clearest for chromosome 1, where CENH3 was concentrated into a single band at one chromosome end, but the spindle microtubules attached along the entire chromosome (**Figures 3B, C**). On the other hand, the observed spindle attachment patterns confirmed the holocentric nature of the *C. europaea* chromosomes, which was also supported by the anaphase arrangement of chromosomes parallel to the equatorial plane of the cell division, which is typical of holocentromeres (**Supplementary Movie 3**). In *C. japonica*, the microtubules attached to chromosomes exclusively at CENH3 chromatin domains (**Figure 3D**).

**Figure 4:**
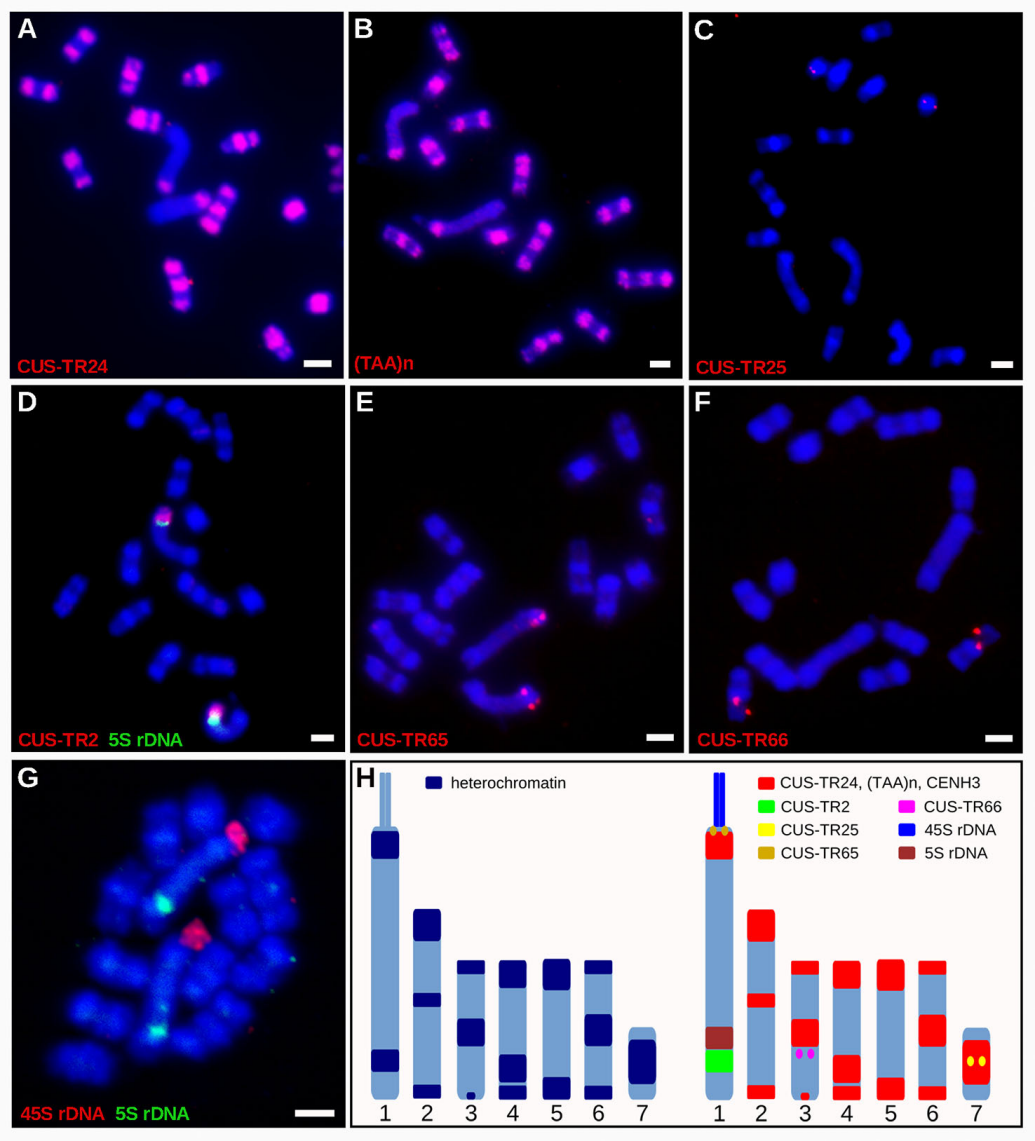
Distribution of satDNA families on chromosomes of *C. europaea*, investigated by FISH. **(A)** CUS-TR24, **(B)** (TAA)n, **(C)** CUS-TR25, **(D)** CUS-TR2 and 5S rDNA, **(E)** CUS-TR65, **(F)** CUS-TR66, **(G)** 45S and 5S rDNAs. Note that 45S rDNA localized to a chromatin tail protruding from one terminus of the largest chromosome 1. The presence of the tail helped to distinguish the CENH3-containing (proximal to the tail) and CENH3-lacking (distal from the tail) heterochromatin domains on this chromosome. Chromosome morphology, size, and repeat distribution patterns allowed us to distinguish all seven chromosome types in *C. europaea*. **(H)** Ideograms summarizing distribution of heterochromatin domains (left), and satDNA families and rDNA repeats (right). CUS-TR67 is not included because it was difficult to detect and its distribution could not be precisely determined. Scale bars = 2 µm.

**Figure 5:**
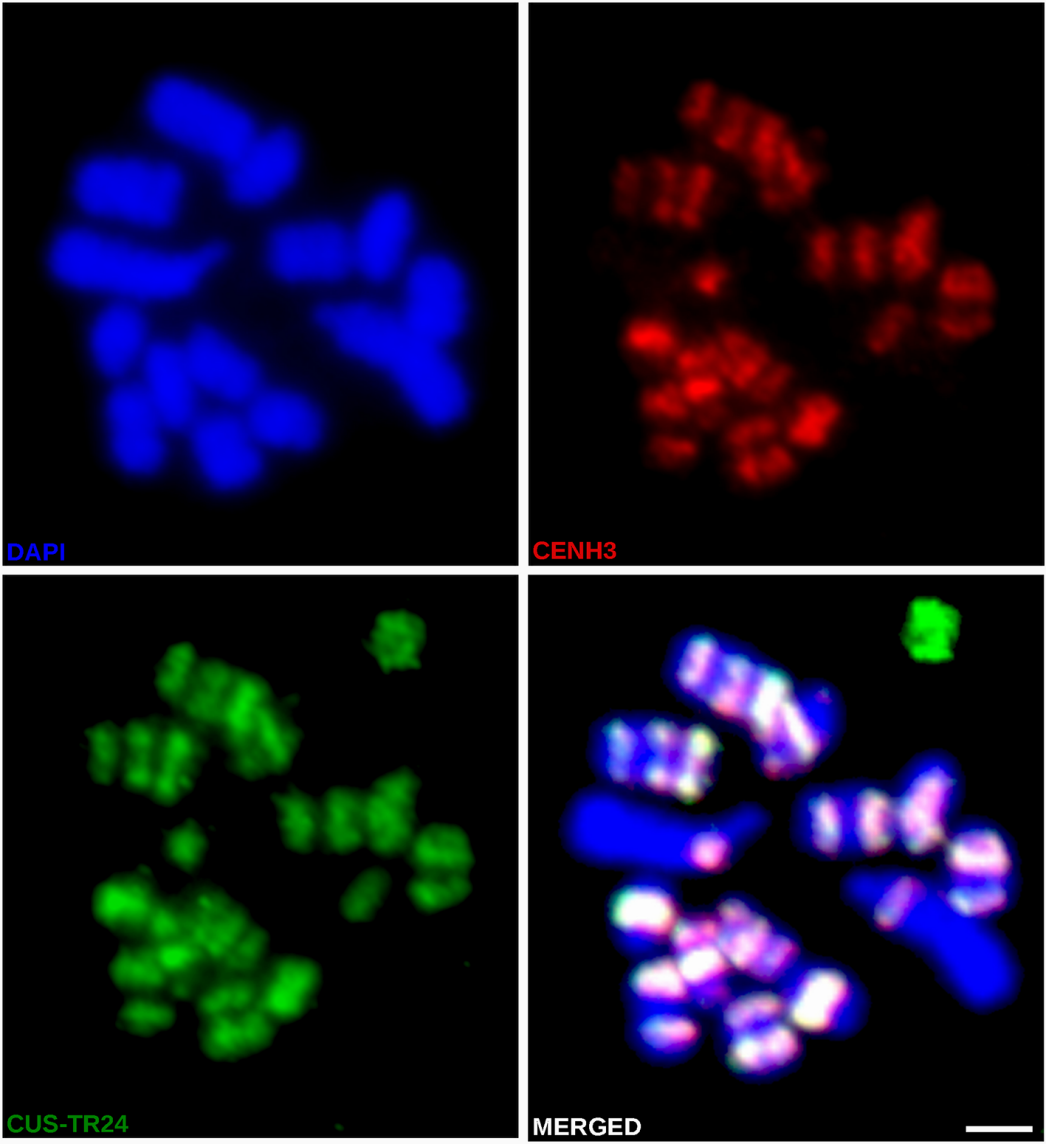
Simultaneous immuno-FISH detection of CENH3 and CUS-TR24 on metaphase chromosomes of *C. europaea*. CENH3 (red) was detected using mixed antibodies against CENH3^CEURO-1a/b^ and CENH3^CEURO-2^. CUS-TR24 and DAPI-stained chromosomes are shown in green and blue, respectively. Scale bar = 2 µm.

In light of the association of CENH3 with heterochromatin, we next sought to determine whether the CENH3 distribution correlated with the presence of specific families of repetitive sequences. To identify such sequences in the *C. europaea* genome, we performed low-pass Illumina sequencing of genomic DNA followed by repeat characterization from the Illumina reads using the RepeatExplorer (Novák et al., 2013) and TAREAN (Novák et al., 2017) pipelines. A particular abundance of satellite DNA (satDNA), constituting 18% of the genome, was found beside all other major types of repetitive sequences (**Supplementary Table 2**). Although six satDNA families and one abundant microsatellite were identified (**Table 2**), this high proportion of satellite DNA was mainly due to the amplification of a single family, CUS-TR24, which alone made up 15.5% of the genome. Because satDNA is a typical constituent of heterochromatic bands on plant chromosomes, we localized all identified tandem repeats, along with rRNA genes, to test for their presence in the DAPI-positive bands (**Figure 4**). Fluorescence *in situ* hybridization (FISH) experiments revealed that all but one of these heterochromatic bands contained the CUS-TR24 satellite (**Figure 4A**), along with the (TAA)n repeat (**Figure 4B**). The same hybridization sites were also associated with the CENH3 chromatin (**Figure 5**). An additional minor satellite repeat, CUS-TR25, was located within the CUS-TR24/CENH3 band on chromosome 7 (**Figure 4C**). The remaining prominent DAPI band on chromosome 1 that was free of CENH3 consisted of satellite CUS-TR2 (**Figure 4D**). The other three satellites were detected as minor loci, apart from the heterochromatic bands (**Figures 4E, F** and data not shown), as schematically depicted in **Figure 4H**.

**Table 2:** Characteristics of satellite DNA families identified in *C. europaea*.

	Genome proportion [%]^a^	TAREAN confidence	monomer length [bp]	proportion of AT [%]
CUS-TR24	15.51	low	389	66.2
CUS-TR2	1.66	high	170	64.8
(TAA)n	0.68^a^	–	3	100.0
CUS-TR65	0.04	high	1714	69.1
CUS-TR25	0.02	high	173	74.5
CUS-TR66	0.02	high	1047	70.6
CUS-TR67	0.01	low	322	71.8

a Genome proportions were estimated from the proportion of reads in the respective RepeatExplorer cluster relative to the total number of reads. The exception was the (TAA)_n_ microsatellite, which was calculated using TRF and TRAP.

## Discussion

In this work, we demonstrated that *C. europaea* chromosomes can be classified as holocentric, based on the attachment of the mitotic spindle microtubules along the entire chromosome length, the absence of primary constrictions, and the orientation of sister chromatids during cell division. This is in agreement with previous reports on species from the same subgenus *Cuscuta*, which were deemed holocentric based on their chromosome morphology and behavior during mitosis and meiosis (Pazy and Plitmann, 1991; Pazy and Plitmann, 1994; Pazy and Plitmann, 1995). However, the distribution of CENH3 proteins on *C. europaea* chromosomes did not correspond to the patterns found in other holocentrics in either the plant or animal kingdoms. Contrary to the known role of CENH3 as a foundational kinetochore protein in most eukaryotes (Westermann and Schleiffer, 2013; Cheeseman, 2014; Hara and Fukagawa, 2017), its atypical confinement into one to three loci per chromosome did not impact the holocentromere-typical arrangement of the mitotic spindle microtubules. Instead, the microtubules also attached to chromosomes at sites where CENH3 was not detected, and their density was not higher at sites of CENH3 accumulation. In contrast to other holocentric plants of genera *Luzula* and *Rhynchospora*, where the holocentromeres form a longitudinal groove extending over almost the entire sister chromatids (Heckmann et al., 2011; Marques et al., 2015; Wanner et al., 2015), the chromosome-spindle interface in *C. europaea* mainly had a smooth surface (**Supplementary Movie 4**).

Assuming that the mitotic spindle binds to chromosomes exclusively at sites where kinetochores are formed (Cheeseman, 2014), our observations suggest that none of the CENH3 variants is an integral component of mitotic kinetochores in *C. europaea*. This contrasts with the notion that CENH3 is essential for kinetochore formation and function (Hara and Fukagawa, 2017). Rare exceptions to this notion include holocentric insect species that lack the *CENH3* genes, implying that they evolved a CENH3-independent mechanism of kinetochore assembly (Drinnenberg et al., 2014; Mon et al., 2017). We speculate that this might also have taken place in *C. europaea*, except that, in this case, the CENH3 genes were preserved and continue to be expressed. An alternative explanation for the discrepancy between the arrangement of microtubules and the distribution of CENH3 is that, in *C. europaea*, these proteins are actually not restricted to sites where they were detected, but also occur in small domains below the limits of microscopy, which are scattered along the entire chromosome length. Theoretically, the kinetochore can assemble on a centromere unit as small as a single CENH3-containing nucleosome, as is the case for point centromeres in *Saccharomyces cerevisiae* (Westermann et al., 2007); therefore, the presence of small CENH3-dependent kinetochores cannot be excluded. However, on the assumption that the stoichiometry between the CENH3, other kinetochore proteins, and attached microtubules is preserved along the chromosome, this hypothesis would predict the higher density of microtubules in CENH3-enriched than in CENH3-depleted regions, which is not in accordance with our observations. Yet another explanation of our results is that CENH3-dependent and CENH3-independent pathways of mitotic kinetochore assembly co-exist in *C. europaea*, the former acting in most heterochromatin domains, and the latter in other parts of the chromosomes. Some of these explanations may be tested by simultaneous localization of other constitutive centromere proteins like CENP-X (Cheeseman, 2014; Hara and Fukagawa, 2017) that may be sufficient to direct kinetochore assembly. Another important question to be addressed in the future experiments is CENH3 distribution and organization of *C. europaea* chromosomes in meiosis, which was not addressed in the present study.

The occurrence of CENH3 exclusively in heterochromatin domains that contain satDNA families (CUS-TR24, CUS-TR25, and (TAA)_n_) resembles the arrangement in most monocentric species, as well as in the holocentric *Rhynchospora pubera*, in which CENH3 is associated mainly with satDNA (Ugarković, 2009; Marques et al., 2015). Although the causes of this phenomenon are not yet well understood, the colocalization of CENH3 with the satDNA families in *C. europaea* suggests that the factors behind the satDNA amplification in CENH3-containing chromatin still are, or were until recently, also active in this species. It is not yet known, however, which DNA sequences constitute the CENH3-lacking holocentromeres in *C. europaea*, but it is clear that arrays of satDNA are not involved, as none of the satDNA families present in this species exhibited a chromosome-wide distribution reflecting the distribution of spindle attachment sites. Thus, the only holocentric organisms in which the centromere domains form on specific repetitive DNA sequences are the *Rhynchospora* species (Marques et al., 2015; Ribeiro et al., 2017). On the other hand, this is not surprising because centromeres are supposed to be determined epigenetically, and although they are usually found in highly repetitive regions, centromeric repeats are neither necessary nor sufficient for centromere specification (McKinley and Cheeseman, 2016).

## Data Availability Statement

The datasets generated for this study can be found in the Sequence Read Archive: ERR3651372 and ERR3651373, GenBank: MN625517-MN625524.

## Author Contributions

PN, AH, and JM conceived the study and designed the experiments. LO, T-SJ, and SK performed the cytogenetics experiments and conventional fluorescence microscopy. AK performed RT-PCR and 3'RACE experiments. PN analyzed the sequenced data. VS carried out the super-resolution microscopy. PN and JM wrote the manuscript with input from LO, T-SJ, SK, AK, VS, and AH. All authors read and approved the final manuscript.

## Funding

This research was financially supported by grants from the Czech Science Foundation (17-09750S) and the Czech Academy of Sciences (RVO:60077344).

## Conflict of Interest

The authors declare that the research was conducted in the absence of any commercial or financial relationships that could be construed as a potential conflict of interest.

## Abbreviations

FISH, fluorescence *in situ* hybridization; RT-PCR, reverse transcription - polymerase chain reaction; 3′RACE, rapid amplification of 3′ cDNA ends; SIM, structured illumination microscopy.

## Supplementary Material

Supplementary Table 1 Primers used for RT-PCR and RACE reactions.

Supplementary Table 2 Proportions of major types of repetitive DNA in the genome of *C. europaea*.

Supplementary Data S1 Sequences of the FISH probes in FASTA format.

Supplementary Movie 1 Three-dimensional organization of CENH3 and tubulin on metaphase chromosomes of *C. europaea*. A two-dimensional image of these chromosomes is shown in **Figure 3**. CENH3 and tubulin are shown in red and green, respectively.

Supplementary Movie 2 Organization of CENH3 and tubulin on metaphase chromosomes of *C. europaea*. The movie plays through optical sections that were acquired for the metaphase shown in **Figure 3** and **Supplementary Movie 1**. CENH3 and tubulin are shown in red and green, respectively.

Supplementary Movie 3 Orientation of *C. europaea* chromosomes during anaphase. The chromosomes are oriented in parallel to the plane of mitotic division, indicating that the pulling force of the mitotic spindle is evenly distributed along the chromosomes.

Supplementary Movie 4 Surface rendering of the DAPI-stained *C. europaea* metaphase chromosomes shown in **Supplementary Movie 1** indicates the absence of distinct structures associated with CENH3-positive chromatin and the microtubule attachment sites. The chromosomes surfaces were produced *via* the surface rendering tool of the Imaris 8.0 software.
