# Supplementary material for: Mitotic Spindle Attachment to the Holocentric Chromosomes of Cuscuta europaea Does Not Correlate With the Distribution of CENH3 Chromatin
Source: Front Plant Sci. 2020 Jan 24;10:1799. doi: 10.3389/fpls.2019.01799 (PMC6992598; doi:10.3389/fpls.2019.01799)
Supplement: Supplementary Data S1 — Sequences of the FISH probes in FASTA format. [file DataSheet_1.pdf]

```
>CUS-TR2 (synthesized oligonucleotide probe labeled at 5' end with biotin)
CCTGAGTGAAAGACGTATATCTTTGTTTCTATTAATAATTTGGACTTGAGTCC

>CUS-TR25  (synthesized oligonucleotide probe labeled at 5' end with biotin)
ACTTAAAATACTAAATGAAGGTTACGTATTACACAAACAAAACCCCGTTGACTTCAAAAC

>CUS-TR24 (synthesized oligonucleotide probe labeled at 5' end with FITC)
AGTGTCACAAATACTTAGCCTTATCTCTATGATTTAGCGTTTTCAGCGAA

>CUS-TR65 (it was amplified from C. europaea genomic DNA using PCR with primers
TCAAACCTTAAGATAAATGGTATTTG and GGCCTTTGCAAAAAATATACA)
AACTACTATTTTTCTTCACTGCTTTTAAATGTAAAATTTTAAAAAGGTAAACATAATAAA
GCTTAACTGGAAAATAACCTCATGATTTGCAAGAAACGAAGTATGATCCGTGTTTGAAAA
AATAAAAACCAAACCTTGGTCAAATAAATATGAGTGAATTTCAGAGACTAGTAAATAGTT
GTTTCAGTAATTAAGAAGAAATCTCTAGGCTCTTAACCCAATAAATTCAACCAAATACCA
TTTATCTTAAGGTTTGA

>CUS-TR66 (it was amplified from C. europaea genomic DNA using PCR with primers
ACTATGCAATTTTCAAAAGCG and GAGCTTCAAGTACCACATTTCA)
AGCTTCAAGTACCACATTTCATCCCCTCGGAAATTTAAATTACACTAAATGCATCAACAT
AAAAGAGTATATAAGCACGTGTAACCACAAGAGATTATTATAAAGCACTACCCAATTTTA
GAGATGTTGAAACATGTATATGTACATCCAGCTAACAAGTTAACACATTAAAAAGTTAAA
GTAAAAGAAGCTAAGCATAACATAGAGCCATATTCAGTCATGTCTGGATAAAAGAGAATG
GATGTAGTATAAATTAAGTGTATAGTTCTTTTAATTTTTCTATAAACTTATCGACTATGC
ATGTTCACTAATAACATCAAAATCCATAAAATTTGAAGCTAAATTGACACATATTTTAAT
ATCAAGTATGATTGGATCTTGTATTTGCTTTTATCACATTTTATACATTAATCACTAAAG
TGACTTAAGCTTTATTAAAATAACATGGGTTATTAAGTTAATCTAGCCTATTTTCTGGCT
ATGAAGCCATTTCTTCCTAATGTTATTTAATATTAAATTTAAATACTTTAAAATAACCAT
AGGAAAATTAAAGTAATGGATAAGATATTAAGACACTTTTTCAAGCCTAAACCTGCCTAG
GCTTTAAAGACTTGTCACGTTTTACGTCTTTTAGAACATTAATTGTACTTGAATAATCAA
CGTACTACTAGAGAATGAGCTTTTCACGTTGGTTATTTTGACATTTTCACGTCGGTTTTG
TATGTGACGTGTAAGTGAGAGACGTTATCGATATAAACATTTTACGTCGGTTCTAAAACC
GACGTGAAAGGTGTATTTTTGAAAGAAAAAAATTAATATACCATGTCGGTTCTTATACAA
AACCGACGTCAAAAGTGTATTTTCGAAAAATAAAATAAATCTCTCACGTCAGTTCTTACA
AAACACCGACGTCAAAAGTATTTTTAATATATATACCACGTCGCTTTCGTTCAATAACCG
TCGTGATAAGTCCATATTTTAAAACAAATATGTGTATTATGTAGTAATATATGCATACAC
GCTTTTGAAAATTGCATAGT

>CUS-TR67 (it was amplified from C. europaea genomic DNA using PCR with primers
CACCATCCCATGCTAGAGA and GAGTGAACTGCATTTTTTGAAGT)
CACCATCCCATGCTAGAGAGTAGTATTTGACATTGTTTTGGATTTCTATGATGAATATAA
TACACAAATTAAAGGAACTCATGTGATGTAATGAAAACTGATTGTTTTGAACTTGGGTTG
TTAATTTTTAATGTTTTTAATCTGCTTTAGTATCTATTAGATTGAGTTGAAGTTTATCAT
TTTCAACTATGCTTTTCACTTCTCAGAATAAGAACTTTTGAGCTTGGGTTGTTTATTTTT
AAAGTTTGAATAACATTTGAAAAATTCCTTTTAAGCTTTTCATTAGATAGAACTACTACT
TCAAAAAATGCAGTTCACTC

>TAAn (synthesized oligonucleotide probe labeled at 5' end with biotin)
TAATAATAATAATAATAATAATAATAATAATAATAATAATAA

>45SrDNA (clone_ID1646; fragment of 45S rDNA amplified using PCR from genomic
DNA of Pisum sativum)
CTAAACCCAGCTCACGTTCCCTATTGGTGGGTGAACAATCCAACACTTGGTGAATTCTGC
TTCACAATGATAGGAAGAGCCGACATCGAAGGATCAAAAAGCAACGTCGCTATGAACGCT
TGGCTGCCACAAGCCAGTTATCCCTGTGGTAACTTTTCTGACACCTCTAGCTTCAAATTC
CGAAGGTCTAAAGGATCGATAGGCCACGCTTTCACGGTTCGTATTCGTACTGGAAATCAG
AATCAAACGAGCTTTTACCCTTTTGTTCCACACGAGATTTCTGTTCTCGTTGAGCTCATC
TTAGGACACCTGCGTTATCTTTTAACAGATGTGCCGCCCCAGCCAAACTCCCCACCTGAC
AATGTCTTCCGCCCGGATCGACCAACCAAAGTCAGCCTTGGATCCAAAAAGAGGGGCAGC
GCCCCGCCTCCGATTCACGGAATAAGTAAAATAACGTTAAAAGTAGTGGTATTTCACTTT
CGCTGTTTCCAGCTCCCACTTATCCTACACCTCTCAAGTCATTTCACAAAGTCGGACTAG
AGTCAAGCTCAACAGGGTCTTCTTTCCCCGCTGATTCCGCCAAGCCCGTTCCCTTGGCTG
TGGTTTCGCTGGATAGTAGACAGGGACAGTGGGAATCTCGTTAATCCATTCATGCGCGTC
ACTAATTAGATGACGAGGCATTTGGCTACCTTAAGAGAGTCATAGTTACTCCCGCCGTTT
```

ACCCGCGCTTGGTTGAATTTCTTCACTTTGACATTCAGAGCACTGGGCAGAAATCACATT
GCGTCAACATCCGCAGGGACCATCGCAATGCTTTGTTTTAATTAAACAGTCGGATTCCCC
TTGTCCGTACCAGTTCTGAGTTGACTGTTCGATGCCCGGGGAAGAGGCCCCGAAAGGCCC
GTTCCCAATCCGTCCCCCGACCGGCACGCGGCGACCCGCTCTCGCCGCGGGAGCAGCTCA
AGCAGTCCACCAACAGCCGACGGGTTCGGAACTGGGACCCCCGTGCCCAGCCCTCAGAGC
CAATCCTTTTCCCGAGGTTACGGATCCATTTTGCCGACTTCCCTTGCCTACATTGTTCCA
TCGACCAGAGGCTGTTCACCTTGGAGACCTGATGCGGTTATGAGTACGACCGGGCATGGA
AGGCACTCGGTCCTCCGGATTTTCAAGGGCCGCCAGGGGCGCACCGGACACCACGCGACG
TGCGGTGCTCTTCCAGCCGCTGGACCCTACCTCCGGCTGAGCCGTTTCCAGGGTGGGCAG
GCTGTTAAACAGAAAAGATAACTCTTTCCGGGGCCCCCGCCAACGTCTCCGGACTCCCTA
ACGTTGCCGTCAGCCACCACGTCCCGGTTCAGGAATTTTAACCCGATTCCCTTTCGGTGT
ACGCGCTTAGAGCGCTATCAGACGGGCTTCCCCCGTCCCTTAGGATCGACTAACCCATGT
GCAAGTGCCGTTCACATGGAACCTTTCCCCTCTTCGGCCTTCAAAGTTCTCATTTGAATA
TTTGCTACTACCACCAAGATCTGCACCGACGACCGCTCCGCCCAGGCTCGCGCCCTGGGT
TTTGCAGCGACCGCCGCGCCCTCCTACTCATCAGGGCTTGGCCCTTGCCCCAACGGCCGG
GTATAGGTCGCGCGCTTTAGCGCCATCCATTTTCGGGGCTAGTTGATTCGGCAGGTGAGT
TGTTACACACTCCTTAGCGGATTTCGACTTCCATGACCACCGTCCTGCTGTCTTAATCGA
CCAACACCCTTTGTGGGGTCTAGGTTAGCGCGCAGTTGGGCACCGTAACCCAGCTTCCGG
TTCATCCCGCATCGCCAGTTCTGCTTACCAAAAATGGCCCACTTGGAGCTCTCGATTCCA
TGGCATGGCTCAACAGAGCAGCCACACCGTCCTACCTATTTAAAGTTTGAGAATAGGTCG
AGGGCGTTGCGCCCCCGATGCCTCTAATCATTGGCTTTACCCGATAGAACTCGCCCTCGG
GCTCCAGCTATCCTGAGGGAAACTTCGGAGGGAACCAGCTACTAGACGGTTCGATTAGTC
TTTCGCCCCTATACCCAAGTCAGACGAACGATTTGCACGTCAGTATCGCTGCGGGCCTCC
ACCAGAGTTTCCTCTGGCTTCGCCCCGCTCAGGCATAGTTCACCATCTTTCGGGTCCCGA
CAGGTATGCTCTCACTCGAACCCTTCACAAAAGATCAGGGTCGGTCGGCGGTGCAACCCA
CAAGAGGATCCCACCAATCAGCTTCCTTGCGCCTTACGGGTTTACTCGCCCGTTGACTCG
CACACATGTCAGACTCCTTGGTCCGTGTTTCAAGACGGGTCGAATGGGGAGCCCACAGGC
CGACGCCAGGAGCACGCAAGTGCCGAAGCACGCCGAATCAGCGCGCGCTGCCGTCCACAA
TCGTGATGATGACGTCTCCGCGAGCATTTCAACAACCCAGGCTTGGGCCACCATCACAAT
CCGCGTCGGTCAATGTCTCGAGTCTATTGGCGGACCGGCACAAACCGTTCCACATCCGAC
CGAGACACATCGCCGGCCCCCATCCGCTTCCCTCCCGACAATTTCAAGCACTCTTTGACT
CTCTTTTCAAAGTCCTTTTCATCTTTCCCTCGCGGTACTTGTTCGCTATCGGTCTCTCGC
CAATATTTAGCCTTGGACGGAATTTACCGCCCGATTGGGGCTGCATTCCCAAACAACCCG
ACTCGCCGACAGCGCCTCGTGGTGCGACAGGGTCCGAGCACAACGGGGCTCTCACCCTCT
CCGGCGCCCCCTTCCAGGGGACTTGGGCCCGGTCCGCCGCTGAGGACGCTTCTCCAGACT
ACAATTCGAACGCCGAGGGCGATCGATTCTCATGGTGGGCTTATCCCGGTTCGCTCGCCG
TTACTAAGGGAATCCTTGTTAGTTCCTTTTCCTCCGCTTATTGATATGCTTAAATTCAGC
GGGTAGCCCCGCCTGACCTGAGGTCTCATCACGAGCGTTTAGAAACGCAAATGGGTAAAA
GAGCCCAAATTCAATAGAGCAACACATGATTGGTCTCGTGGGTCACACAACCACCATTTA
TCATGGCACACCCTACCAAGGTCTCAATTTTCAACCAACCATGAGACGAAAGAGAGCTCA
CGGGAGGCCAACATCCACCCTGCACAATACCTGTCAAAAGGAAATTGGCAGGAGGCTTCA
ATATGTGACACCCAGGCAGACGTGCCCTCAACCTAATGGCATCGGGCGCAACTTGCGTTC
AAAGACTCGATGGTTCACGGGATTCTGCAATTCACACCAAGTATCGCATTTCGCTACGTT
CTTCATCGATGCAAGAGCCTAGATATCCGTTGCCGAGAGTCATTTTATATTAATGTGTCA
AAACACAACCCGCACGGGAACCGTCTCCGGTGCCATGCAGATGTGCCCAGAGCAAAGTTA
AATTTCCTTGACGCATTCAGCGTCGGGGTTTGAGTTTTGGCACAGAGGAGAACGCATTTT
GTCGTTCCCCTCCGATGCCAATGGCAAGCTGGGGTGTGAAACACCTCAAGCCCACCGCAT
GTGTTCAAACTAATTCACGTGTTGGACTGCATATAAGGCATCGACAATGATCCTTCCGCA
GGTTCACCTACGGAAACCTTGTTACGACTTCTCCTTCCTCTAAATGATAAGGTTCAGTGG
ACTTCTCACAACGTCGCGGGCAGCGAACCGCCCACGTCGCCGCAATCCGAACACTTCACC
GGACCATTCAATCGGTAGGAGCGACGGGCGGTGTGTACAAAGGGCAGGGACGTAGTCAAC
GCGAGCTGATGACTCGCGCTTACTAGGAATTCCTCGTTGAAGACCAACAATTGCAATGAT
CTATCCCCATCACGATGAAATTTCAAAGATTACCCGGGCCTGTCGGCCAAGGCTATAGAC
TCGTTGAATACATCAGTGTAGCGCGCGTGCGGCCCAGAACATCTAAGGGCATCACAGACC
TGTTATTGCCTCAAACTTCCGTGGCCTAAGCGGCCATAGTCCCTCTAAGAAGCTGGCCGT
GGAGGGTTACCTCCACATAGCTATTTAGCAGGCTGAGGTCTCGTTCGTTAACGGAATTAA
CCAGACAAATCGCTCCACCAACTAAGAACGGCCATGCACCACCACCCATAGAATCAAGAA
AGAGCTCTCAGTCTGTCAATCCTTACTATGTCTGGACCTGGTAAGTTTCCCCGTGTTGAG
TCAAATTAAGCCGCAGGCTCCACTCCTGGTGGTGCCCTTCCGTCAATTCCTTTAAGTTTC
AGCCTTGCGACCATACTCCCCCCGGAACCCAAAGACTTTGATTTCTCATAAGGTGCCAGC
GGAGTCCTAAAAGCAACATCCGCTGATCCCTGGTCGGCATCGTTTATGGTTGAGACTAGG
ACGGTATCTGATCGTCTTCGAGCCCCAACTTTCGTTCTTGATTAATGAAAACATCCTTG
GCAAATGCTTTCGCAGTTGTTCGTCTTTCATAAATCCAAGAATTTCACCTCTGACTATGA
AATACGAATGCCCCCGACTGTCCCTGTTAATCATTACTCCGATCCCGAAGGCCAACACAA

TAGGATCAGAATCCTGTGGTGTTATCCCATGCTAATGTATCCAGAGCGTAGGCTTGCTTT
GAGCACTCTAATTTCTTCAAAGTAACAGCGCCGGAGGCACGACCCAGCCAATTAAGGCCA
GGAGCGCATCGCCGGCAGAAGGGACGAGCCAACCGGTGCACACCAGAGGCGGACCGATCA
ACCCAACCCAAGGTCCAACTACGAGCTTTTTAACTGCAACAACTTAAATATACGCTATTG
GAGCTGGAATTACCGCGGCTGCTGGCACCAGACTTGCCCTCCAATGGATCCTCGTTAAGG
GATTTAGATTGTACTCATTCCAATTACCAGACTCAATGAGCCCGGTATTGTTATTTATTG
TCACTACCTCCCCGTGTTAGGATTGGGTAATTTGCGCGCCTGCTGCCTTCCTTGGATGTG
GTAGCCGTTTCTCAGGCTCCCTCTCCGGAATCGAACCCTAATTCTCCGTCACCCGTCACC
ACCATGGTAGGCCACTATCCTACCATCGAAAGTTGATAGGGCAGAAATTTGAATGATGCG
TCGCCAGCACAAAGGCCGTGCGATCCGACGAGTTATCATG

>5SrDNA (clone_ID1761; fragment of 5S rDNA amplified using PCR from genomic DNA of Pisum sativum)
GCGATCATACCAGCACTAATGCACCGGAACCCATCAGAACTCCGCAGTTAAGCGTGCTTG
GGCGAGAGTAGTACTAGGATGGGTGACCTCCTGGGAAGTCCTTGTGTTGCACCTCTTGTT
TTGGGATTTTTTAAATACTTGTATCTATTTTTGTTAATCGTCGTAAAAGAATGGAAAGAA
GTAGGAGAAAACAAATATTTCAGCGTTAATTATGTCTGTTCTGCCGTTAATAAGTGA
